# Correlation between fat-to-muscle mass ratio and cognitive impairment in elderly patients with type 2 diabetes mellitus: a cross-sectional study

**DOI:** 10.1186/s12877-024-04941-2

**Published:** 2024-04-18

**Authors:** Fan Wu, Yanlan Liu, Chenying Lin, Nahal Haghbin, Longfei Xia, Yaoshuang Li, Tong Chen, Huina Qiu, Weiran Jiang, Jingbo Li, Jingna Lin

**Affiliations:** 1 Department of Endocrinology, Tianjin Union Medical Center, Nankai University Affiliated Hospital, Tianjin, China; 2 Tianjin Union Medical Center, Tianjin Medical University, Tianjin, China; 3 School of Global Health, Chinese Center for Tropical Diseases Research, Shanghai Jiao Tong University School of Medicine, Shanghai, China; 4 Tianjin Union Medical Center, School of Medicine, Nankai University, Tianjin, China; 5 Eastman Institute for Oral Health, University of Rochester Medical Center, Rochester, NY USA

**Keywords:** Fat-to-muscle mass ratio, Cognitive impairment, Type 2 diabetes mellitus, Sex, Body composition

## Abstract

**Background:**

Fat to muscle mass ratio (FMR), a novel index integrating fat and muscle composition, has garnered attention in age-related conditions such as type 2 diabetes mellitus (T2DM) and neurodegenerative diseases. Despite this research on the relationship between FMR and cognitive impairment (CI) in T2DM remains scarce. This study aimed to investigate the sex-specific association between FMR and CI in elderly T2DM patients.

**Methods:**

A total of 768 elderly (> 60 years) T2DM in-patients (356 men and 412 women) were recruited from the Department of Endocrinology at Tianjin Nankai University affiliated hospital. Bioelectrical Impedance Analysis (BIA) was used to assess body composition, and Montreal Cognitive Assessment (MoCA) was used to evaluate cognitive performance. T2DM patients were categorized into normal cognitive function (NC) and cognitive impairment (CI) groups based on MoCA scores and stratified by sex. Binary logistic regression was employed to examine the association between FMR and CI.

**Results:**

Among the participants, 42.7% of men and 56.3% of women experienced cognitive deterioration. Women with CI exhibited lower body mass index (BMI) and skeletal muscle mass index (SMI), while men with cognitive disorders showed lower SMI, FMR, and higher fat mass index (FMI). FMR was consistently unrelated to cognition in females, irrespective of adjustment made. However, in males, FMR was significantly associated with an increasing risk of cognitive dysfunction after adjusting for demographic and clinical variables (OR: 1.175, 95% *CI*: 1.045–1.320, *p* = 0.007). Furthermore, for each 0.1 increase in FMR, the incidence of CI rose by 31.1% after additional adjustment for BMI. In males, the prevalence of CI increased sequentially across FMR quartiles (*p <* 0.05).

**Conclusion:**

Elderly T2DM men with high FMR had unfavorable cognitive function. FMR is independently associated with an increased risk of CI in male T2DM patients regardless of BMI.

## Introduction

In the elderly population diagnosed with type 2 diabetes mellitus (T2DM), cognitive impairment (CI) had emerged as a significant concern, representing a prominent feature of neurodegenerative conditions. As the aging demographic expands, CI, being a prevalent age-associated condition, has garnered considerable research interest. Epidemiological data shows that dementia prevalence among individuals aged ≥ 60 years in China stands at 6.0%, with mild cognitive impairment (MCI) affecting 15.5% [1]. Moreover epidemiological studies suggest that T2DM heightens the risk of dementia compared with age-matched non-diabetic subjects [2, 3]. Concurrently, diabetic patients with cognitive dysfunction face challenges in effectively self-manage chronic disease, thereby exacerbating the progression of diabetes, thus initiating a vicious cycle [4].

Obesity is an independent risk factor for diabetes [5] prevalent among T2DM patients aged 60 and above. Historically, obesity has been considered as a hazardous factor for cognitive function [6], with excess adipose linked to neurocognitive decline. However, recent studies have presented conflicting findings, with an increasing number failing to establish a direct correlation between obesity and dementia risk, and some even suggesting the opposite. Interestingly, CI has been associated with weight loss in older adults [7]. The influence of obesity on cognitive function may be obscured by the widespread use of body mass index (BMI) as a measure of obesity. In older adults, a high BMI may paradoxically serve as a protective factor for cognitive function, perhaps due to the limitations of BMI in distinguishing between fat mass and muscle mass. Besides a higher BMI indicates better nutritional status, which is conducive to preserve cognitive function [8].Therefore there arises a need to identify new indicators capable of distinguishing between fat and muscle mass.

Fat-to-muscle mass ratio (FMR), a novel index derived from the composition of fat and muscle, has emerged as a promising alternative for assessing the imbalance of muscle and fat [9]. Research indicates that FMR serves as a valuable indicator of metabolic and inflammatory status [10], exhibiting significant correlations with insulin resistance and metabolic syndrome [11], both of which are associated with CI. While a growing number of studies have investigated the role of FMR in cognitive dysfunction, the majority have focused on the general community population [12, 13], leaving a notable gap in research pertaining to patient with T2DM. In this article, we endeavor to elucidate the relationship between FMR and CI in elderly individuals with T2DM, aiming to offer lifestyle recommendations that integrate considerations of both muscle and fat components to enhance cognitive function.

## Materials and methods

### Study design and participants

We conducted a cross-sectional study involving adult subjects aged 60 years and above who had T2DM and attended the Department of Endocrinology in Tianjin Nankai University Affiliated Hospital between July 2018 and May 2022.

### Inclusion and exclusion criteria

#### Inclusion criteria

(I) Diagnosis of T2DM in individuals according to the diagnostic criteria for diabetes mellitus outlined by the World Health Organization (WHO) in 1999 [14];(II) Age above 60 years; (III) Sufficient visual and auditory abilities to undergo neuropsychological test and body composition measurement.

#### Exclusion criteria

(I) Inability to complete neuropsychological scale screening due to speech impairment, hearing loss or reluctant to cooperate; (II) Inability to undergo bio-electrical impedance analysis due to conditions such as edema, paralysis, installed pacemaker, with metal objects insides or reluctant to cooperate; (III) Presence of conditions potentially affecting cognitive function, including head trauma, cerebral ischemia, mental and neurological disorders such as depression, anxiety, delirium, dementia and drug addiction; (IV) Concurrent presence of anemia, severe lung or kidney disease, history of heart failure, malignant tumors, hypothyroidism, hyperthyroidism etc.; (V) Repeat hospitalization.

### Body composition

Body composition analysis adapts direct segmental multifrequency Bioelectrical Impedance Analysis (BIA) (InBody720; Biospace Co, Ltd, Seoul, Korea). Subjects were asked to place their five fingers on the surface of the electrode, with their heels and forefeet covering the round electrode. During the measurement, the subject should try to avoid shaking and touching other parts of the body. The measurements were recorded by well-trained staff and completed within 30s. Weight, fat mass and skeletal muscle mass were obtained from the test. BMI = weight (kg) / height^2^ (m^2^); Fat Mass Index (FMI) = fat mass (kg) / height^2^ (m^2^); Skeletal Muscle mass Index (SMI) = skeletal muscle mass (kg) / height^2^ (m^2^); FMR = fat mass (kg) / skeletal muscle mass (kg).

### Screening evaluation for cognitive impairment

Global cognitive function was assessed using the Montreal Cognitive Assessment (MoCA) test. The MoCA test demonstrates a sensitivity of 90% for detecting MCI in older adults with [15]. Higher MoCA scores indicate better cognition, with a threshold score of 25/26 indicating the presence of MCI [16]. Additionally, for participants with 12 years of education or fewer, one point was added to his/ her total MoCA score to calibrate the bias of education level [15].

In this study, we divided the subjects into Normal Cognitive Function (NC) group: MoCA ≥ 26; and Cognitive Impairment (CI) group:MoCA < 26.

### Covariates

Participants completed a face-to-face questionnaire regarding their demographic characteristics, including sex, age, years of education, drinking and smoking status, insulin therapy, metformin treatment etc. Marital status was classified as married or single. Regular exercise was defined as engaging in moderate-to-intense physical activity for more than 150 min per week [17]. Diabetic diet control refers to adhering to the dietary recommendations in the Expert Consensus on Diagnosis and Treatment measures for Chinese Elderly Patients with T2DM [18]. Prior history of diseases was provided by self-report and medical records, including hypoglycemia in last three months, diabetic nephropathy, cardiovascular disease and cerebrovascular disease. Blood pressure is measured by a professional using a standard upper arm type electronic blood pressure monitor (Omron, HBP-9021).

Subjects were required to fast overnight for at least 8 h for further serum biochemical analysis. Indicators including Fast Plasma Glucose (FPG), Total Cholesterol (TC), Triglycerides (TG) were tested by an automatic biochemical analyzer (TBA-120FR, Toshiba, Japan), while hemoglobin A1c (HbA1c) was analyzed using a fully automated Glycohemoglobin analyzer (HA-8180, ARKRAY, Japan).

### Statistical analysis

The baseline characteristics with continuous variables were presented as mean ± standard deviation or median (interquartile range) according to the consequence of normal distribution test and categorical variables as an absolute number and percentage (%) of the total. Student’s t test was used for comparison of normally distributed data between the two groups, and Mann Whitney U test was used for comparison of non-normally distributed data. Categorical data were analyzed by chi-square test. Because the body composition of males and females are significantly different, our analysis further conducted stratified by sex.

To evaluate the correlation between FMR and CI, logistic regression was adopted to calculate the Odds Ratio (*OR*) and 95% Confidence Interval (*CI*) in multiple models. Model 1 adjusted for age, years of education; Model 2 further adjusted for being married, dietary control of diabetes, smoking, diabetic nephropathy, cerebrovascular disease, systolic blood pressure (SBP), HbA1c and metformin. Logistic regression analysis was conducted to determine the correlations between FMR quartiles and the risk of CI. For quartiles 2–4, the *OR* and 95% *CI* of cognitive impairment were calculated and compared with the lowest quartile as the reference category stratified by sex. Statistical analysis was conducted by SPSS version 25.0 with a significance level set at a two-tailed *p* value of < 0.05 for all tests.

## Result

### Baseline characteristics

**Fig. 1:**
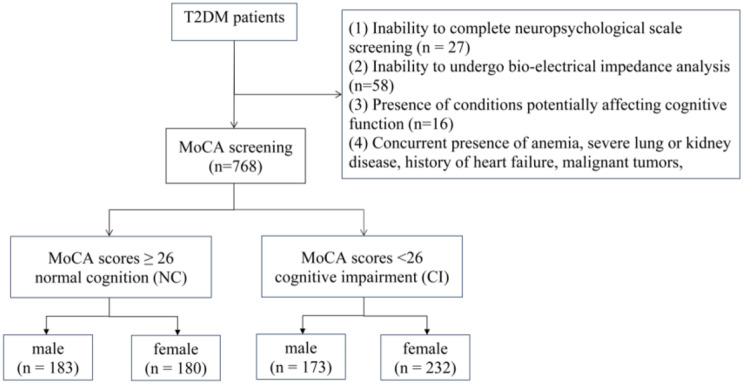
Flow chat of study participants

Figure 1 described the flow chart of our study. Among the 927 subjects diagnosed with T2DM, 768 subjects were ultimately enrolled in the study based on the exclusion criteria. This group comprised 356 men (46.4%, mean age 66.7) and 412 women (mean age 67.1). The characteristics of participants grouped by cognitive status are displayed in Table 1. Participants with CI tended to be older, female, solitude, and had lower level of education, less dietary control, a higher prevalence of cerebrovascular disease, lower SMI (9.34 ± 1.11 vs. 9.70 ± 1.11, *p* < 0.001) and higher FMR (0.93 ± 0.31 vs. 0.86 ± 0.31, *p* = 0.008) compared to those with NC. Other demographic characteristic like smoking, drinking, SBP, diastolic blood pressure (DBP) and regular exercise, as well as insulin therapy, metformin treatment, and medical history dates such as hypoglycemia, diabetic nephropathy and cardiovascular disease, along with laboratory indexes including TC, TG, FBG and HbA1c, showed no difference between two groups. Given the substantial differences in cognition and body composition between sexes, we further stratified the analysis by sex and cognitive status, as shown in Table 2. Among men, 173 (42.7%) subjects suffered from CI, while among women, 232 (56.3%) were affected. Notably, the difference in age and dietary between two groups was not statistically significant among men. However, male patients with CI showed a higher prevalence of smoking and a lower prevalence of metformin treatment, whereas female with CI exhibited a higher prevalence of smoking and diabetic nephropathy. As for body composition, women with CI had a lower BMI (25.88 ± 3.28 vs. 26.64 ± 2.82, *p* = 0.031), with a notable reduction in SMI (8.78 ± 0.89 vs. 9.03 ± 0.85, *p* = 0.005), while indices such as FMI and FMR did not demonstrate meaningful differences. Conversely, men with CI shown lower SMI (10.10 ± 0.93 vs. 10.36 ± 0.93, *p* = 0.009), higher FMI (7.42 ± 2.37vs. 6.86 ± 2.03, *p* = 0.018) and higher FMR (0.73 ± 0.22 vs. 0.66 ± 0.19, *p* = 0.001), with no significant difference observed in BMI between the groups.

**Table 1:** Characteristics of the study population by cognition status

Characteristics	NC (*n* = 363)	CI (*n* = 405)	P
Male	183 (50.4%)	173 (42.7%)	0.033*
Age (years)	66.3 ± 4.8	67.4 ± 5.0	0.004*
Education (years)	10.8 ± 2.7	9.8 ± 2.9	< 0.001*
Smoking	131 (36.1%)	167 (41.2%)	0.144
Drinking	113 (31.1%)	116 (28.6%)	0.452
Married	331 (91.2%)	330 (81.5%)	< 0.001*
SBP (mmHg)	135.3 ± 12.9	134.0 ± 13.2	0.166
DBP (mmHg)	79.14 ± 8.2	79.2 ± 9.1	0.848
Regular exercise	268 (73.8%)	283 (69.9%)	0.225
Dietary control of diabetes	269 (74.1%)	264 (65.2%)	0.007*
Hypoglycemia	92 (25.3%)	111 (27.4%)	0.517
Diabetic nephropathy	131 (36.1%)	171 (42.2%)	0.082
Cerebrovascular disease	99 (27.3%)	170 (42.0%)	< 0.001*
Cardiovascular disease	154 (42.4%)	177 (43.7%)	0.721
Insulin therapy	158 (43.5%)	179 (44.2%)	0.852
Metformin	161 (44.4%)	178 (44.0%)	0.911
TC (mmol/l)	4.8 (4.1, 5.5)	5.7 (4.0, 5.5)	0.347
TG (mmol/l)	1.4 (1.1, 2.0)	1.4 (1.1, 1.9)	0.389
FBG (mmol/l)	7.6 (6.2, 9.9)	7.8 (6.4, 9.5)	0.835
HbA1c (%)	8.1 (7.1, 9.8)	8.4 (7.2, 10.2)	0.254
Height(cm)	165.74 ± 8.07	164.33 ± 8.35	0.018*
Weight(kg)	71.95 ± 11.24	70.02 ± 11.74	0.021*
FM (kg)	22.61 ± 7.27	22.96 ± 6.86	0.490
SMM (kg)	26.88 ± 5.01	25.48 ± 5.05	< 0.001*
BMI (kg/m^2^)	26.15 ± 3.41	25.85 ± 3.29	0.225
FMI (kg/m2)	8.31 ± 2.89	8.56 ± 2.67	0.221
SMI (kg/m2)	9.70 ± 1.11	9.34 ± 1.11	< 0.001*
FMR	0.86 ± 0.31	0.93 ± 0.31	0.008*
MoCA	, 27 (26, 28)	23, (22, 24)	< 0.001*

Data are presented as means ± standard deviations or medians (interquartile ranges) for continuous data and numbers (%) for categorical data. *p < 0.05. Abbreviation: NC: normal cognitive; CI: cognitive impairment; SBP: systolic blood pressure; DBP: diastolic blood pressure; TC: total cholesterol; TG: triglycerides; FPG: fasting plasma glucose; HbA1c: hemoglobin A1c; FM: fat mass; SMM: skeletal muscle mass; BMI: body mass index; FMI: fat mass index; SMMI: skeletal muscle mass index; FMR: fat to muscle mass ratio; MoCA: Montreal cognitive assessment

**Table 2:** Characteristics of the study population by cognition status and sex

Characteristics	Men(*n* = 356)			Women(*n* = 412)		
	NC (*n* = 183)	CI (*n* = 173)	*p*	NC (*n* = 180)	CI (*n* = 232)	*p*
Age (years)	66.3 ± 5.1	67.1 ± 5.5	0.195	66.3 ± 4.6	67.6 ± 5.2	0.007*
Education(years)	11.2 ± 2.9	10.4 ± 2.5	0.007*	10.5 ± 2.5	9.4 ± 3.2	< 0.001*
Smoking	119 (65.0%)	131 (75.7%)	0.027*	12 (6.7%)	36 (15.5%)	0.005*
Drinking	109 (59.6%)	108 (62.4%)	0.580	4 (2.2%)	8 (3.4%)	0.463
Married	176 (96.2%)	154 (89.0%)	0.009*	155 (86.1%)	176 (75.9%)	0.009*
SBP (mmHg)	135.3 ± 13.7	134.4 ± 14.1	0.532	135.3 ± 12.3	133.8 ± 12.5	0.193
DBP (mmHg)	79.1 ± 8.3	80.1 ± 8.4	0.270	79.2 ± 8.2	78.6 ± 9.6	0.548
Regular exercise	135 (73.8%)	124 (71.7%)	0.657	133 (73.9%)	159 (68.5%)	0.235
Dietary control of diabetes	134 (73.2%)	113 (65.3%)	0.106	135 (75.0%)	151 (65.1%)	0.030*
Hypoglycemia	43 (23.5%)	41 (23.7%)	0.964	49 (27.2%)	70 (30.2%)	0.512
Diabetic nephropathy	80 (43.7%)	80 (46.2%)	0.632	51 (28.3%)	91 (39.2%)	0.021*
Cerebrovascular disease	57 (31.1%)	74 (42.8%)	0.023*	42 (23.3%)	96 (41.4%)	< 0.001*
Cardiovascular disease	74 (40.4%)	74 (42.8%)	0.655	80 (44.4%)	103 (44.4%)	0.992
Insulin therapy	84 (45.9%)	85 (49.1%)	0.542	74 (41.1%)	94 (40.5%)	0.903
Metformin	89 (48.6%)	66 (38.2%)	0.046*	72 (40.0%)	112 (48.3%)	0.094
TC (mmol/l)	4.6 ± 1.1	4.6 ± 1.2	0.373	5.0 ± 1.1	5.0 ± 1.1	0.641
TG (mmol/l)	1.5 (1.1, 1.9)	1.3 (1.0, 1.8)	0.605	1.5 (1.1, 2.2)	1.5 (1.2, 2.1)	0.393
FBG (mmol/l)	8.0 (6.4, 10.1)	7.9 (6.4, 9.4)	0.750	7.5 (6.2, 9.4)	7.5 (6.2, 9.3)	0.883
HbA1c (%)	8.2 (7.1, 10.1)	8.3 (7.2, 10.1)	0.757	7.8 (7.0, 9.5)	8.4 (7.2,10.1)	0.187
Height(cm)	171.88 ± 5.29	171.51 ± 5.60	0.517	159.49 ± 5.07	158.98 ± 5.61	0.337
Weight(kg)	75.93 ± 9.80	76.13 ± 11.77	0.864	67.90 ± 11.19	65.47 ± 9.45	0.020*
FM (kg)	20.27 ± 6.00	21.86 ± 7.27	0.026*	24.98 ± 7.69	23.78 ± 6.43	0.093
SMM (kg)	30.66 ± 3.59	29.80 ± 3.83	0.028*	23.02 ± 2.92	22.26 ± 3.05	0.011*
BMI (kg/m2)	25.67 ± 2.89	25.82 ± 2.32	0.645	26.64 ± 2.82	25.88 ± 3.28	0.031*
FMI (kg/m2)	6.86 ± 2.03	7.42 ± 2.37	0.018*	9.80 ± 2.89	9.42 ± 2.56	0.157
SMI (kg/m2)	10.36 ± 0.93	10.10 ± 0.93	0.009*	9.03 ± 0.85	8.78 ± 0.89	0.005*
FMR	0.66 ± 0.19	0.73 ± 0.22	0.001*	1.08 ± 0.28	1.07 ± 0.29	0.895
MoCA	27, (26, 28)	23, (22, 24)	< 0.001*	27, (26, 28)	23, (22, 24)	< 0.001*

Data are presented as means ± standard deviations or medians (interquartile ranges) for continuous data and numbers (%) for categorical data. *p < 0.05. Abbreviations: NC: normal cognitive; CI: cognitive impairment; SBP: systolic blood pressure; DBP: diastolic blood pressure; TC: total cholesterol; TG: triglycerides; FPG: fasting plasma glucose; HbA1c: hemoglobin A1c; FM: fat mass; SMM: skeletal muscle mass; BMI: body mass index; FMI: fat mass index; SMMI: skeletal muscle mass index; FMR: fat to muscle mass ratio; MoCA: Montreal cognitive assessment

### Association of FMR with cognitive performance

The associations between FMR and CI in multivariate-adjusted models among men and women are presented in Table 3. Initially, in unadjusted logistic regression analyses, a significant association was observed within the male group. Even after adjusting for age and years of education, the association persisted. Furthermore, when additional adjustments were made for other demographic variables, medical history, and biochemical parameters, the significance of the association remained unchanged in male (*OR*: 1.175, 95% *CI*: 1.045–1.320, *p* = 0.007). To evaluate the impact of BMI on this risk relationship, we further adjusted for BMI and found that for every 0.1 increase in FMR, the risk of CI increased by 31.1%. Notably, BMI appeared to have a borderline protective effect on cognition. Conversely, among female, irrespective of the indicators adjusted, FMR consistently demonstrated no significant association with cognition. However, higher BMI levels were associated with a lower risk of CI (*OR*: 0.882, 95% *CI*: 0.805–0.967, *p* = 0.007).

**Table 3:** Logistic regression analyses of the association of FMR with CI by sex

FMR	OR	95% CI	p
**Male**			
Unadjusted	1.187	1.068–1.320	0.002*
Model 1	1.175	1.053–1.312	0.004*
Model 2	1.175	1.045–1.320	0.007*
Model 3	1.311	1.104–1.558	0.002*
BMI	0.907	0.813–1.013	0.083
**Female**			
Unadjusted	0.995	0.929–1.066	0.894
Model 1	0.989	0.922–1.062	0.764
Model 2	0.989	0.917–1.067	0.774
Model 3	1.107	0.989–1.239	0.077
BMI	0.882	0.805–0.967	0.007*

*OR*s and *p* values were estimated for each 0.1 increase in FMR. *p < 0.05. Model 1 adjusted for age, years of education; Model 2 further adjusted for married, dietary control of diabetes, smoking, diabetic nephropathy, cerebrovascular disease, systolic blood pressure, hemoglobin A1c, and metformin; Model 3 further adjusted for BMI

### FMR quartile

**Fig. 2:**
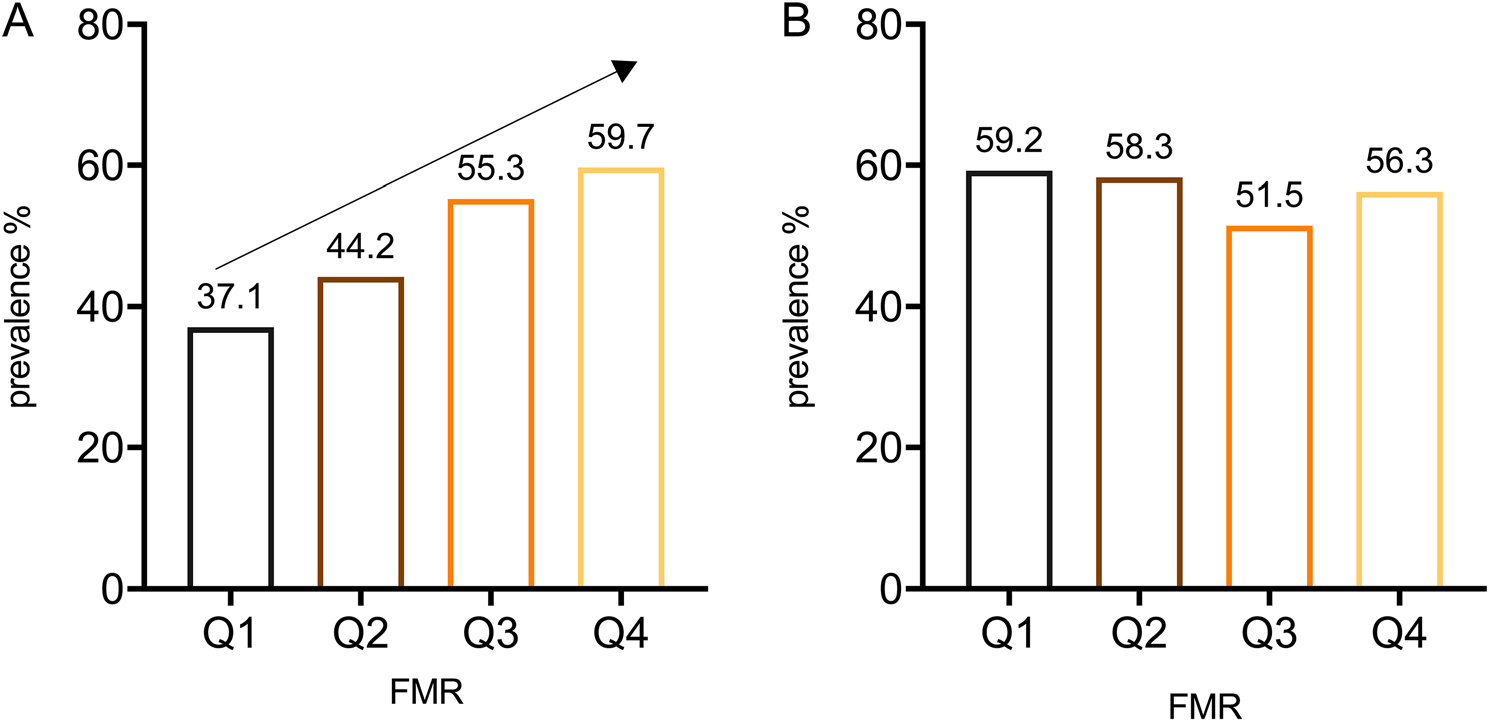
Quartile of FMR and prevalence of CI in patients with T2DM

Figure 2 illustrates a sequential increase in the prevalence of CI from the lowest to the highest quartiles of FMR in male (*p* < 0.05). Compared to individuals in the lowest quartile (Q1) of FMR, those in the highest quartile (Q4) exhibited a significantly elevated risk of cognitive decline in Model1 (*OR*: 2.178, 95% *CI*: 1.101–4.312, *p* < 0.05) (Table 4). Even after further adjusted for BMI in model 2, the highest quartile (Q4) of FMR remained associated with a significant higher risk of CI (*OR*: 2.980,95% *CI*: 1.88–7.473, *p* < 0.05) among males. Conversely, in the female group, irrespective of whether it was Model 1 or Model 2, there was consistently no discernible relationship between FMR and CI. However, BMI continued to exhibit a protective effect, consistent with the findings mentioned above.

A for male, *p* for trend < 0.05.B for female, *p* for trend ≥ 0.05.

**Table 4:** Logistic regression analyses of the association of FMR quartile with CI stratified by sex

Quartile of FMR	Male		Female	
	N(range)	OR (95%CI)	N(range)	OR (95%CI)
**Model 1**				
Q1	89 (< 0.55)	1(ref)	103 (< 0.90)	1(ref)
Q2	89 (0.55–0.66)	1.192 (0.634–2.240)	103 (0.90–1.04)	1.038 (0.563–1.911)
Q3	89 (0.67–0.87)	1.282 (0.682–2.411)	103 (1.05–1.23)	0.945 (0.515–1.737)
Q4	89 (≥ 0.88)	2.178 (1.101–4.312) *	103 (≥ 1.24)	0.870 (0.476–1.592)
*P* for trend		0.032*		0.595
**Model 2**				
Q1	89 (< 0.55)	1(ref)	103 (< 0.90)	1(ref)
Q2	89 (0.55–0.66)	1.325 (0.682–2.573)	103 (0.90–1.04)	1.307 (0.686–2.491)
Q3	89 (0.67–0.87)	1.504 (0.743–3.046)	103 (1.05–1.23)	1.361 (0.686-2.700)
Q4	89 (≥ 0.88)	2.980 (1.188–7.473) *	103 (≥ 1.24)	1.684 (0.738–3.840)
*P* for trend		0.031*		0.241
BMI		0.959 (0.869–1.059)		0.904 (0.830–0.985) *

Model 1 were adjusted for potential confounders including age, years of education, marital status, dietary control of diabetes, smoking, diabetic nephropathy, cerebrovascular disease, SBP, HbA1c, and metformin. Model 2 were further adjusted for BMI. *p < 0.05

## Discussion

This cross-sectional study aimed to investigate the relationship between FMR and cognitive dysfunction in elderly patients with T2DM. Our findings revealed that individuals in the cognitive decline group exhibited higher FMR levels, with FMR emerging as an independent risk factor for CI among elderly men with T2DM. Notably, this association remained significant even after adjusting for potential confounders, including BMI. To our knowledge, this is the first study to explore the relationship between FMR and cognition in T2DM patients.

Several studies have separately investigated the relationship between fat mass, muscle mass, and the risk of CI; however, these findings are not always consistent. A prospective cohort study involving 453 patients found that the decline in SMI over time was independently associated with global cognitive decline in patients with T2DM [19]. Whereas a cross-sectional study in Japan, which included 497 patients with MCI and 684 with Alzheimer’s disease (AD), found a significant correlation between memory function and sarcopenia in women, but not in men [20]. Regarding fat mass, a study of 542 community residents aged 21 to 90 years found a significant association between obesity defined by the first two quintiles of FMI and a greater likelihood of attentional impairment (*OR*: 2.05, 95%*CI* 1.12–3.82) [21]. A study involving elderly obese veterans in Texas found that cognitive decline in older age may worsen with increased fat mass, which appears to be an important predictor of cognitive performance in this population [22]. Conversely, a study involved 5169 patients found that higher abdominal and thigh subcutaneous fat content was associated with a lower likelihood of dementia in women [23]. The reasons for these significant differences in associations are unclear. To comprehensively consider the impact of muscle mass and fat mass on cognitive function, we conducted a study on the FMR and CI.

A prospective study exploring the associations of FMR with dementia risk, regarding dementia subtypes, observed that the association between FMR and AD was significant only in men, which is consistent with this article [13]. However, studies by Ma et al. found that higher FMR was associated with a higher prevalence of cognitive impairment (*OR*: 1.44, 95%*CI*: 0.88–2.35, *p* for trend = 0.029), mainly occurring in women and the elderly [12]. The evidence suggests that the sex-specific effect of FMR on CI risk and the nature of this association are not fully understood. In this study, moderately high FMR was associated with an increased risk of CI in elderly men with T2DM, but not in women. In women, although there was no significant difference between FMR and CI, the BMI and SMI of cognitive decline group were shown lower. In the final model, BMI rather than FMR showed an independent association with CI, consistent with previous research findings in women. The combination of BMI and FMR may highlight the important role of simple muscle loss in female cognition. This may be related to the neuroprotective effect of muscle tissue. Based on studies of body composition index and cognition, we speculate that the aforementioned differences may be related to sex-specific differences in the effects of fat, with the cognitive harm of body fat being more pronounced in men than in women. A recent study based on diffusion-weighted imaging in the UK Biobank cohort found that body fat percentage (PBF) and white matter brain age gap (BAG) were correlated differently between genders. Greater PBF in males was linked to higher BAG, while lower PBF in females was associated with higher BAG [24]. We propose several possible biological hypotheses to explain this: First, fat accumulation in males is mainly manifested as increased visceral fat, while in females it is mainly concentrated in subcutaneous tissue [25]. Visceral adipose tissue has been shown to have higher metabolic activity than subcutaneous adipose tissue and is thought to have a stronger influence on inflammatory cytokines and insulin resistance [26]. Previous research found a direct correlation between visceral obesity, but not subcutaneous fat, and poor cognitive performance in older adults [27]. However, in this article, we did not include visceral fat area as a variable, which can be further improved in future studies. Second, female patients are generally obese, with high fat content and minimal composition change, which do not play an important role in cognitive function [8]. In addition, hormonal differences between men and women may be at the center of the story. Adipose tissue, the main source of estrogen in women after menopause, plays a protective role in cognition [28]. Unlike women, aging in men is often accompanied by decreased testosterone levels. Larger fat tissue may increase levels of aromatase, an enzyme that converts testosterone into estrogen [29]. In men, aromatase may be involved in accelerating endocrine and brain aging processes [30].

In summary, we found that muscle loss plays an important role in women. However, in men, muscle loss and fat accumulation work synergistically. Human body as a whole, with its various components interacting with each other. Some studies have proposed the definition of sarcopenic obesity (SO) as the coexistence of fat accumulation, muscle mass loss and poor function, and comprehensively studied the relationship with cognitive function [31]. Numerous studies have linked SO to severe CI, compared to obesity or sarcopenia [32]. The FMR used in this study has been proposed as a substitute for the evaluation of SO [9], and this paper is consistent with the results of previous studies. Based on the results of this study, we emphasized the difference in the role of fat between sexes and the protective effect of muscle, while also recommending monitoring the distribution of body composition in elderly diabetics and adjusting lifestyle to improve cognitive function.

The study may have several potential limitations. First, it was a cross-sectional study and because of the study design, only correlations, not causation, could be established. Although we adjusted for demographic, lifestyle, and diabetes-related confounders, some factors were not included, such as the Apolipoprotein E4 gene and nutritional status. Second, we use BIA to measure body composition, rather than the gold standard dual-energy X-ray absorption method. However, BIA is closely related to dual-energy X-ray absorption method, which has been used widespread in research and shown reliability in assessing body composition. Ultimately, this was a single-center study with all participants from the same tertiary hospital in North China, so we may not generalize the results to other population groups. Further multicenter, prospective cohort studies are needed.

## Conclusion

Our study underscores the independent relationship between FMR and heightened risk of CI in male patients with T2DM. Higher FMR levels were consistently linked to CI, regardless of BMI. These findings suggest that FMR could serve as a valuable metric in routine health screening and co-management strategies for T2DM patients, with the potential to mitigate the onset of CI.

## Data Availability

The raw data used in this study are available from the corresponding authors: JNL and JBL.

## Abbreviations

T2DM: Type 2 diabetes mellitus
CI: Cognitive impairment
NC: Normal cognitive
MCI: Mild cognitive impairment
BMI: Body mass index
FMR: Fat to muscle mass ratio
WHO: World Health Organization
BIA: Bioelectrical impedance analysis
FMI: Fat mass index
SMI: Skeletal muscle mass index
MoCA: Montreal cognitive assessment
FPG: Fast plasma glucose
TC: Total cholesterol
TG: Triglycerides
HbA1c: Hemoglobin A1c
OR: Odds ratio
95%CI: 95% confidence interval
SBP: Systolic blood pressure
DBP: Diastolic blood pressure
AD: Alzheimer’s disease
PBF: body fat percentage
BAG: brain age gap
SO: Sarcopenic obesity
